# Privacy-Preserving Record Linkage of Deidentified Records Within a Public Health Surveillance System: Evaluation Study

**DOI:** 10.2196/16757

**Published:** 2020-06-24

**Authors:** Long Nguyen, Mark Stoové, Douglas Boyle, Denton Callander, Hamish McManus, Jason Asselin, Rebecca Guy, Basil Donovan, Margaret Hellard, Carol El-Hayek

**Affiliations:** 1 Burnet Institute Melbourne Australia; 2 School of Population Health and Preventive Medicine Monash University Melbourne Australia; 3 Department of General Practice HaBIC Research Technology Unit, Melbourne Medical School University of Melbourne Melbourne Australia; 4 Kirby Institute University of New South Wales Sydney Australia; 5 Mailman School of Public Health Columbia University New York, NY United States; 6 Sydney Sexual Health Centre Sydney Australia; 7 Department of Infectious Diseases The Alfred Hospital Melbourne Australia; 8 Doherty Institute and School of Population and Global Health University of Melbourne Melbourne Australia

**Keywords:** medical record linkage, public health surveillance, sentinel surveillance, sensitivity and specificity, data linkage, confidentiality, evaluation studies as a topic

## Abstract

**Background:**

The Australian Collaboration for Coordinated Enhanced Sentinel Surveillance (ACCESS) was established to monitor national testing and test outcomes for blood-borne viruses (BBVs) and sexually transmissible infections (STIs) in key populations. ACCESS extracts deidentified data from sentinel health services that include general practice, sexual health, and infectious disease clinics, as well as public and private laboratories that conduct a large volume of BBV/STI testing. An important attribute of ACCESS is the ability to accurately link individual-level records within and between the participating sites, as this enables the system to produce reliable epidemiological measures.

**Objective:**

The aim of this study was to evaluate the use of GRHANITE software in ACCESS to extract and link deidentified data from participating clinics and laboratories. GRHANITE generates irreversible hashed linkage keys based on patient-identifying data captured in the patient electronic medical records (EMRs) at the site. The algorithms to produce the data linkage keys use probabilistic linkage principles to account for variability and completeness of the underlying patient identifiers, producing up to four linkage key types per EMR. Errors in the linkage process can arise from imperfect or missing identifiers, impacting the system’s integrity. Therefore, it is important to evaluate the quality of the linkages created and evaluate the outcome of the linkage for ongoing public health surveillance.

**Methods:**

Although ACCESS data are deidentified, we created two gold-standard datasets where the true match status could be confirmed in order to compare against record linkage results arising from different approaches of the GRHANITE Linkage Tool. We reported sensitivity, specificity, and positive and negative predictive values where possible and estimated specificity by comparing a history of HIV and hepatitis C antibody results for linked EMRs.

**Results:**

Sensitivity ranged from 96% to 100%, and specificity was 100% when applying the GRHANITE Linkage Tool to a small gold-standard dataset of 3700 clinical medical records. Medical records in this dataset contained a very high level of data completeness by having the name, date of birth, post code, and Medicare number available for use in record linkage. In a larger gold-standard dataset containing 86,538 medical records across clinics and pathology services, with a lower level of data completeness, sensitivity ranged from 94% to 95% and estimated specificity ranged from 91% to 99% in 4 of the 6 different record linkage approaches.

**Conclusions:**

This study’s findings suggest that the GRHANITE Linkage Tool can be used to link deidentified patient records accurately and can be confidently used for public health surveillance in systems such as ACCESS.

## Introduction

### Background

The Australian Collaboration for Coordinated Enhanced Sentinel Surveillance (ACCESS) of blood-borne viruses (BBVs) and sexually transmissible infections (STIs) monitors diagnostic testing and other episodes of care for priority BBVs and STIs [1,2]. ACCESS extracts deidentified patient data from a network of laboratories and clinics, including those that manage high caseloads of patients from populations with heightened risk for BBVs and STIs, including gay, bisexual, and other men who have sex with men and people who inject drugs. The main objective of ACCESS is to measure and report key indicators such as disease incidence and prevalence (measured as proportion positive) to monitor and support Australia’s efforts to reduce the transmission of morbidity and mortality caused by BBV and STI [3-5].

A key challenge for ACCESS (and similar sentinel surveillance systems) is that patient outcomes can be inaccurately measured if individuals attend multiple health services, leading to potential reporting bias. For example, markers of testing frequency, an important indicator for BBV/STI prevention and management [3-5], may be underestimated if individuals test at multiple services. Accurate linkage of individuals within and between services in ACCESS provides more accurate measures of (1) the key indicators relating to Australia’s BBV and STI National Strategies and (2) the effect of interventions aimed at reducing the impact of BBVs and STIs.

The linkage of deidentified ACCESS records across sites relies on specialized health data extraction software GRHANITE, which is installed at participating clinics and laboratories. GRHANITE interfaces with patient databases, securely extracting line-listed consultation, demographic, BBV and STI clinical and pathology data [6]. Before data are transferred from ACCESS sites, GRHANITE creates a unique record ID to identify an electronic medical record (EMR) and uses patient-identifying information to generate irreversible hash-coded linkage keys associated with that record. The record ID and linkage keys, rather than the personal identifiers, are transferred with the patients’ clinical and pathology data to a secure data bank, preserving patient privacy [7]. A link ID is then generated [8] when the same patient is linked across different EMRs by matching linkage keys using a companion software called the GRHANITE Linkage Tool [9].

### Objectives

The GRHANITE Linkage Tool has been validated to perform large-scale population-level record linkage [10] to achieve similar sensitivity and specificity data linkage profiles as per traditional person-identifiable data linkage mechanisms [9]. Given that there is variation in the available person-identifiable data at clinical and laboratory sites in ACCESS, the focus of this paper is to assess the quality of linkage results by applying different approaches to using the GRHANITE Linkage Tool in ACCESS. To evaluate the GRHANITE Linkage Tool for ongoing public health surveillance, we measured the outcomes of record linkage using the tool against gold-standard linked datasets derived from ACCESS data.

## Methods

### Australian Collaboration for Coordinated Enhanced Sentinel Surveillance Data Extraction and Linkage via GRHANITE

#### Electronic Medical Records

Typically, when a patient first attends a medical facility, an EMR is created in the facility’s patient database, containing the patient’s identifying information, including the name, date of birth, contact details, and Medicare number (an Australian government–issued health care card number used for Medicare billing). Most clinics will also have recorded other demographic information, such as preferred language, country of birth, and indigenous background in the EMR.

Every individual’s EMR has a unique medical record number generated by the patient database, linking all of a patient’s consultations, tests, and prescription records. Multiple EMRs may be created for one patient at the same facility if the patient’s details change and are not updated, leading to the creation of a new EMR; if the patient uses an alias; or if the patient attends a clinic that allows anonymous or free testing.

#### Data Extraction in the Australian Collaboration for Coordinated Enhanced Sentinel Surveillance

**Figure 1:**
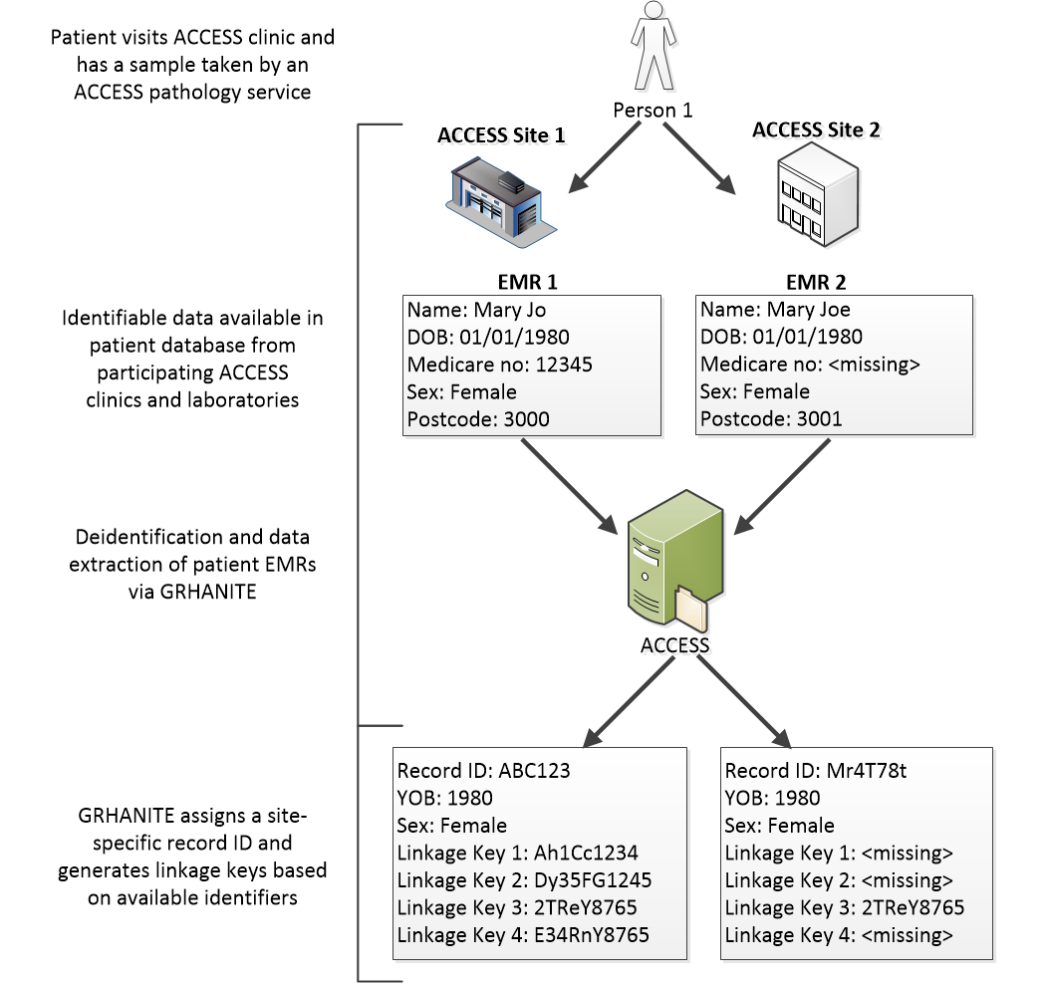
Data extraction in the Australian Collaboration for Coordinated Enhanced Sentinel Surveillance: using GRHANITE to deidentify electronic medical records and create linkage keys.

Data were extracted from participating ACCESS clinical sites that included an EMR for every patient available in their databases at the time of extraction. GRHANITE generated a new unique record ID and up to four irreversible hash-coded linkage keys for each EMR. Personal identifying information (eg, name, date of birth, Medicare number) in the patient’s EMR was passed through advanced encryption to generate both record ID and linkage keys [7]. The record ID and linkage keys were extracted by GRHANITE alongside the patient demographics, consultation, test request, pathology, and prescription information related to BBV and STI care, without the identifying information. Data extraction was similar for laboratories; however, only BBV and STI test records related to diagnosis and care and a limited set of demographic variables were available for extraction (Figure 1).

#### Record Linkage in the Australian Collaboration for Coordinated Enhanced Sentinel Surveillance

The data components used by GRHANITE to create the linkage keys include the following patient identifiers: 5 digits of the Medicare number, date of birth, sex, first name, last name, and residential postcode. However, not all EMRs have the same set of patient identifiers recorded in the same way. For example, a patient name may be recorded as *William* in one clinic with a full date of birth and *Bill* in another clinic with only a year of birth recorded. GRHANITE utilizes data preprocessing to remove unwanted characters and words and to resolve nicknames utilizing an Australian national nickname list. Phonetic encoding (double metaphone) is then employed, which permits fuzzy matching based on misspellings of the surname and forename. Transposition of day and month of birth is also supported. After preprocessing, identifiers are combined and then encrypted utilizing secret seeding keys and cryptographic hashing to generate the GRHANITE privacy-preserving cryptographic hashed linkage keys [7,9].

GRHANITE creates up to four linkage keys for each EMR, using combinations of identifying information that is recorded at each site (Textbox 1) [11]. For example, if the Medicare number was not recorded for a patient, then linkage keys that require 5 Medicare digits (Textbox 1: linkage key types 1, 2, and 4) could not be created, resulting in EMRs extracted via GRHANITE having only one linkage key (Textbox 1: linkage key 3 does not require the Medicare number; Figure 1).

**Types of linkage keys generated by GRHANITE.**

Linkage key and components of base identifying information:

- Type 1: 5 Medicare digits; date of birth; and sex
- Type 2: 5 Medicare digits; postcode; first three characters of first name; and year of birth
- Type 3: Last name and first name (either order permitted) and fuzzy matching used; date of birth with day/month (transpositions permitted)
- Type 4: Last name and first name (either order permitted) and fuzzy matching used; 5 Medicare digits

#### Applying the GRHANITE Linkage Tool

**Figure 2:**
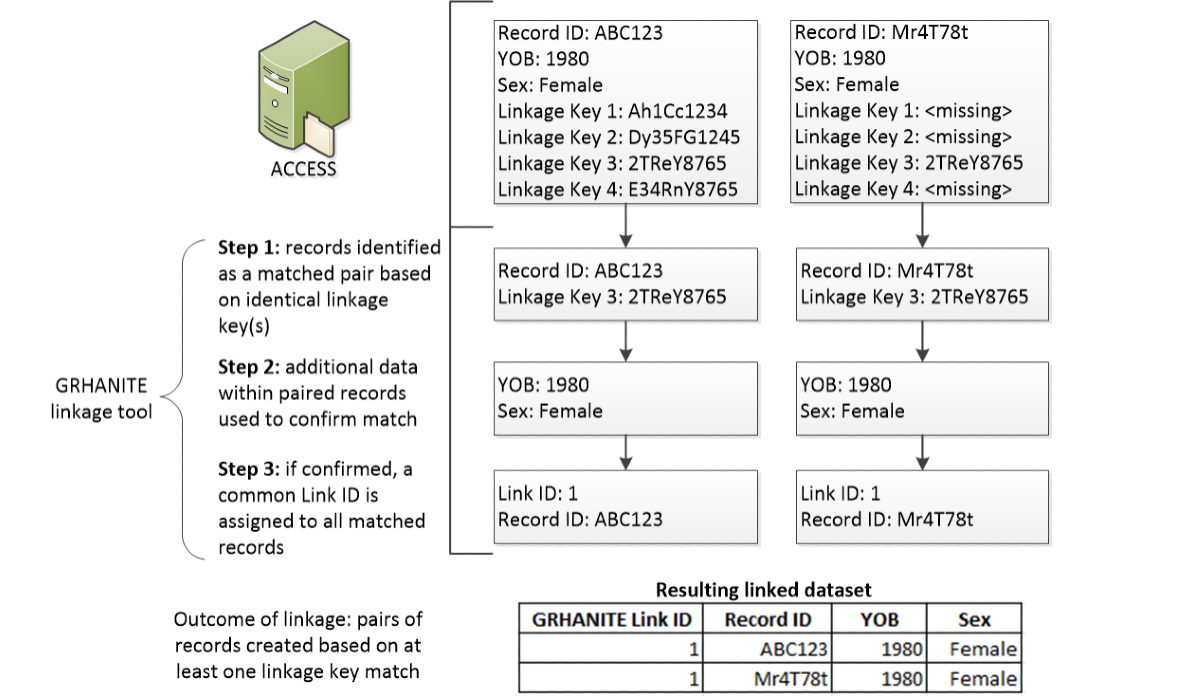
Record linkage in the Australian Collaboration for Coordinated Enhanced Sentinel Surveillance: using the GRHANITE Linkage Tool to identify and accept matches.

There are three steps in the record linkage process in ACCESS when applying the linkage tool. The first step finds pairs of EMRs based on at least one linkage key matching and records the linkage key type/s used to match each record pair. The second step examines the strength of the link using other available data within the matched pair of records to accept or reject linked records as described in Table 1. The third step assigns an identifier (a link ID) to the accepted matched pairs to label all matched records as belonging to the same individual (Figure 2).

**Table 1:** GRHANITE Linkage Tool approaches to accepting matches.

Linkage approach	Description
Accept all	Accept all record links as determined by the linkage tool
Year of birth match	Accept only record links if year of birth matches
Sex match	Accept only record links if sex matches
Year of birth and sex match	Accept only record links if year of birth and sex match
Two or more linkage keys^a^	Accept record links only if matched on two or more linkage key types
Linkage key type 3 plus sex match^b^	Accept only record links that match on linkage key type 3 and match on sex

^a^Given that 3 out of the 4 linkage key types are generated using the Medicare number, this approach requires the Medicare number to be present in the EMR (Textbox 1).

^b^This approach only relies on linkage key type 3, which does not require the Medicare number to be present in the EMR.

### Evaluating the Record Linkage

#### Creating the Gold-Standard Datasets

To evaluate the record linkage in ACCESS, we generated two gold-standard datasets, using a deterministic record linkage method, where the true match status could be identified [12]. To assess the outcomes of the six linkage approaches described in Table 1, using the GRHANITE Linkage Tool for matching the records in the gold-standard datasets, we measured the sensitivity, specificity, and positive and negative predictive values, where possible.

#### The PrEPX Gold-Standard Dataset

**Figure 3:**
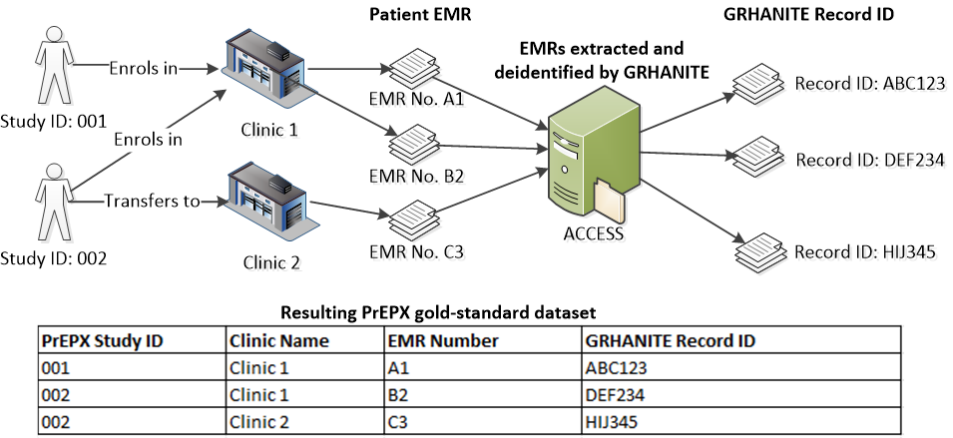
Data flow of electronic medical records in PrEPX and deterministic linkage for the gold-standard dataset.

PrEPX is a population-level intervention study in Victoria in which HIV pre-exposure prophylaxis was made available to eligible individuals, and the study used ACCESS data to monitor participants’ BBV and STI testing [13]. Eight clinical sites and one hospital clinic participating in ACCESS had PrEPX participants enrolled between July 2016 and March 2018. At enrollment, a PrEPX study ID was sequentially assigned and recorded alongside each participant’s enrollment-clinic EMR number in a study database. Following enrollment in PrEPX, if a participant attended a different clinic within the network during the study period, the EMR number from the new clinic was also recorded in the study database. ACCESS had ethics approval to extract the EMR number from the participating clinics for the purpose of matching the records of participants who moved among clinics. To create the gold-standard dataset, EMRs were matched on clinic EMR number, clinic name, and clinic visit date. The gold standard included one record per PrEPX participant who attended only one clinic during the study period and multiple records per PrEPX participant who attended multiple clinics linked by study ID (Figure 3).

#### The Pathology Results Gold-Standard Dataset

**Figure 4:**
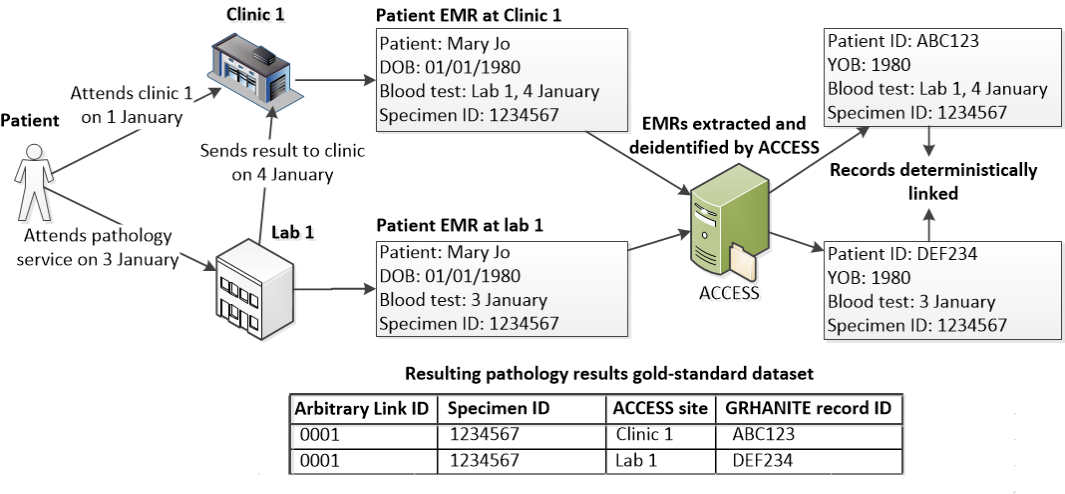
Data flow of pathology results in electronic medical records and deterministic linkage for the gold-standard dataset.

A second and much larger gold-standard dataset was generated from the EMRs extracted from 7 clinics and 4 laboratories participating in ACCESS between January 2009 and April 2018. To be included in this dataset, patients had to have at least one specimen sent from one of the ACCESS clinics to one of the ACCESS laboratories. A unique laboratory specimen ID was assigned to the specimen at the laboratory, and when laboratories returned pathology results to the clinic, this specimen ID was also recorded at the clinic. To create the gold-standard dataset, clinic and laboratory records were matched using the laboratory specimen ID, year of birth, and test date. We allowed for a 7-day difference in test dates, as in medical records, the recorded date can commonly vary for the same specimen. Only matched records were included in the gold-standard dataset and linked using an arbitrarily assigned link identifier (Figure 4).

An EMR in the pathology results gold-standard dataset may match to many other EMRs for several reasons, including the following: individuals may have had multiple specimens sent to multiple laboratories for testing, individuals may have attended different clinics and therefore had the same test result sent from the laboratory to more than one clinic, or individuals may have had multiple EMRs at the laboratory or clinic as a result of outdated or incomplete personal identifiers.

#### Data Analysis

##### Sensitivity

The sensitivity was calculated as the number of correctly linked EMRs, as identified using the GRHANITE Linkage Tool, as a percentage of the total number of linked EMRs in the gold standard dataset.

##### Specificity

In the PrEPX gold-standard dataset, the specificity was calculated as the number of single EMRs correctly identified as unlinked using the GRHANITE Linkage Tool as a percentage of the total number of unlinked EMRs. The positive predictive value (PPV) and negative predictive value were also calculated to provide probabilities of true matches and missed matches.

**Figure 5:**
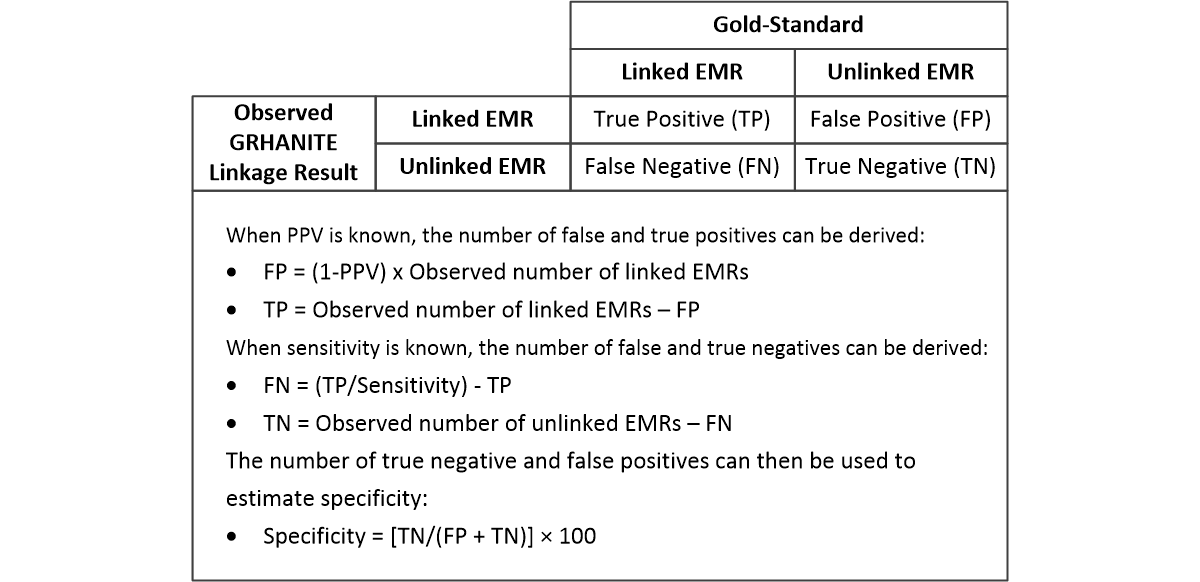
Estimating specificity when positive predictive value and sensitivity are known.

Given the deidentified nature of the ACCESS data, it was not possible to include unmatched specimen IDs in the pathology results gold-standard dataset because there was no way to confirm whether they belonged to different individuals (correctly unmatched), making it impossible to calculate specificity. Therefore, to evaluate specificity, we assessed the concordance of chronological HIV and hepatitis C test records to identify EMRs that should not have been linked. By identifying the linked EMRs with discordant results, the PPV (the proportion of linked records with concordant antibody results) could be determined. The specificity was then estimated using the PPV and the sensitivity for each linkage approach as summarized in Figure 5.

##### Measuring Incorrect Matches Using Discordant Pathology Results

Following infection, any HIV or hepatitis C antibody test that subsequently occurs should always return a positive result. Using the pathology results gold-standard dataset provided only a small number of HIV and hepatitis C results; therefore, a dataset of linked EMRs was derived using all available EMRs from the same clinic and laboratory sites used to create the gold-standard dataset. Two datasets were created, one that contained any HIV western blot or antibody result and one that contained any hepatitis C antibody result. EMRs containing discordant results before record linkage were excluded from the sample so as not to confuse it with discordance resulting from record linkage. Records within each dataset were then linked using all six approaches (Table 1) of the GRHANITE Linkage Tool. Linked EMRs where there was no history of a positive result were removed from the sample, as a discordant test result can only be determined after an initial positive result. Therefore, in the HIV and hepatitis C datasets, only linked EMRs that contained an antibody result after an initial positive HIV western blot or hepatitis C antibody result were retained for evaluation.

To calculate the PPV, the linked EMRs were then inspected for negative antibody results occurring at least seven days after a positive test result, which were then classified as incorrectly matched. Where most subsequent antibody tests were negative, the initial and any subsequent positive results were considered incorrectly matched records.

## Results

### Record Linkage Using the PrEPX Gold-Standard Dataset

The PrEPX gold-standard dataset identified 28 joins among 56 EMRs, indicating 28 study participants had attended two different clinical sites during the PrEPX study period. The remaining 3644 EMRs were from participants who only attended a single clinic during the study and therefore did not have any linked records.

Over 99% of EMRs had all four linkage key types present in 8 of the 9 sites, indicating that the patient-identifying information to generate those linkage keys was near fully recorded at the clinics. One site was missing data needed to generate linkage types 1, 2, and 4 (which all require the Medicare number) in 11% (8/76) of their EMRs (Table 2).

In all linkage approaches, except the approach requiring two or more linkage keys, all pairs of EMRs from the 28 individuals who attended two sites were correctly joined (100% sensitivity). With the approach which required two or more linkage keys for matching, one pair was not identified (96% sensitivity). Specificity was 100% using all linkage approaches, without any of the remaining 3644 EMRs in the dataset being falsely linked (Table 3).

**Table 2:** Percentage of electronic medical records in the PrEPX gold-standard dataset by linkage key type and site.

Site	Number of electronic medical records, N	Percentage of electronic medical records with Linkage Key			
		Type 1, n (%)	Type 2, n (%)	Type 3, n (%)	Type 4, n (%)
Site 1	76	68 (89)	68 (89)	76 (100)	68 (89)
Site 2	853	853 (100.0)	853 (100.0)	853 (100.0)	853 (100.0)
Site 3	1087	1087 (100.00)	1084 (99.72)	1087 (100.00)	1087 (100.00)
Site 4	582	582 (100.0)	582 (100.0)	582 (100.0)	582 (100.0)
Site 5	40	40 (100.0)	40 (100.0)	40 (100.0)	40 (100.0)
Site 6	135	135 (100.0)	135 (100.0)	135 (100.0)	135 (100.0)
Site 7	106	106 (100.0)	103 (99.2)	106 (100.0)	106 (100.0)
Site 8	314	314 (100.0)	314 (100.0)	314 (100.0)	314 (100.0)
Site 9	507	507 (100.0)	507 (100.0)	507 (100.0)	507 (100.0)
Total	3700	3692 (99.78)	3686 (99.62)	3700 (100.00)	3692 (99.78)

**Table 3:** Evaluation measures derived from using the GRHANITE Linkage Tool on the PrEPX gold-standard dataset.

Linkage approach	Sensitivity (N=56), n (%)	Specificity (N=3644), n (%)	Positive predictive value (N=56), n (%)	Negative predictive value (N=3644), n (%)
Accept all	56 (100)	3644 (100.00)	56 (100)	3644 (100.00)
Year of birth match	56 (100)	3644 (100.00)	56 (100)	3644 (100.00)
Sex match	56 (100)	3644 (100.00)	56 (100)	3644 (100.00)
Year of birth and sex match	56 (100)	3644 (100.00)	56 (100)	3644 (100.00)
Two or more linkage keys	54 (96)	3644 (100.00)	54 (100)^a^	3644 (99.90)^b^
Linkage key type 3 plus sex match	56 (100)	3644 (100.00)	56 (100)	3644 (100.00)

^a^N=54.

^b^N=3646.

### Record Linkage Using the Pathology Results Gold-Standard Dataset

Using the GRHANITE Linkage Tool on the pathology results gold-standard dataset created 50,484 linked records among 86,538 EMRs, with a maximum of six EMRs identified as belonging to the same individual.

A total of 99.69% (86,273/86,538) of EMRs contained at least one linkage key type, and all four linkage key types were present in 73.51% (63,610/86,538) of records, suggesting that the completion of patient-identifying information in the patient database was very high overall. However, 21.62% (18,709/86,538) of EMRs had only linkage key type 3 available for matching. One or more of linkage types 1, 2, and 4 (which all require the Medicare number) was missing in 97.42% (7914/8124) of EMRs from one public laboratory, 53.95% (5967/11,060) of EMRs from the sexual health clinic, 48.25% (1403/2908) of EMRs from a private laboratory, and 23.42% (6134/26,186) of EMRs from another public laboratory (Table 4).

For the first 4 linkage approaches, the GRHANITE Linkage Tool correctly linked 94% to 95% of EMRs in the pathology results gold-standard dataset, dropping to 66% (57,330/86,538) where two or more linkage keys are needed to form a match (Table 5). In the final linkage approach, where pairs were only accepted when matched on linkage key type 3 (which does not require the Medicare number) and sex, 89% (76,928/86,538) of records were correctly linked.

**Table 4:** Percentage of electronic medical records in the pathology gold-standard dataset by linkage key type and site.

Site	Number of electronic medical records, N	Number of electronic medical records with no linkage keys, n (%)	Percentage of electronic medical records with Linkage Key			
			Type 1, n (%)	Type 2, n (%)	Type 3, n (%)	Type 4, n (%)
Clinic 1	3165	0 (0.00)	3083 (97.41)	3077 (97.22)	3165 (100)	3083 (97.41)
Clinic 2	6342	0 (0.00)	6031 (95.10)	6015 (94.84)	6342 (100)	6031 (95.10)
Clinic 3	2514	0 (0.00)	2493 (99.16)	2489 (99.01)	2513 (99.96)	2492 (99.12)
Clinic 4	9679	0 (0.00)	9351 (96.61)	9322 (96.31)	9676 (99.97)	9350 (96.60)
Clinic 5	1369	1 (0.07)	1357 (99.12)	1356 (99.05)	1368 (99.93)	1357 (99.12)
Clinic 6	2489	5 (0.20)	2315 (93.01)	2288 (91.92)	2484 (99.80)	2315 (93.01)
Clinic 7 (sexual health)	11,060	9 (0.08)	5097 (46.08)	5094 (46.06)	11,049 (99.90)	5095 (46.07)
Lab 1 (public)	26,186	241 (0.92)	23,705 (90.53)	20,059 (76.60)	25,465 (97.25)	23,227 (88.70)
Lab 2 (public)	8124	8 (0.10)	215 (2.65)	210 (2.58)	8116 (99.90)	215 (2.65)
Lab 3 (private)	2908	1 (0.03)	1706 (58.67)	1509 (51.89)	2907 (99.97)	1710 (58.80)
Lab 4 (private)	12,702	0 (0.00)	12,205 (96.09)	12,203 (96.07)	12,700 (99.98)	12,203 (96.07)
Total	86,538	265 (0.31)	67,558 (78.07)	63,622 (73.52)	85,785 (99.13)	67,078 (77.51)

**Table 5:** Evaluation measures derived from using the GRHANITE Linkage Tool on the pathology results gold-standard dataset.

Linkage approach	Gold standard (N=86,538)	HIV results			Hepatitis C results		
	Sensitivity, n (%)	N	Positive predictive value, n (%)	Estimated specificity, (%)	N	Positive predictive value, n (%)	Estimated specificity, (%)
Accept all	82,345 (95.15)	1427	1245 (87.25)	90.52	3908	3866 (98.93)	99.32
Year of birth match	82,212 (95.00)	1412	1234 (87.39)	90.71	3817	3777 (98.95)	99.34
Sex match	81,689 (94.40)	1257	1143 (90.93)	93.20	3810	3775 (99.08)	99.42
Year of birth and sex match	81,560 (94.25)	1263	1152 (91.21)	93.42	3775	3741 (99.10)	99.43
Two or more linkage keys	57,330 (66.25)	257	256 (99.6)	99.74	2809	2795 (99.50)	99.67
Linkage key type 3 plus sex match	76,928 (88.90)	1090	984 (90.28)	92.98	3626	3596 (99.17)	99.49

### Estimating Specificity Using Discordant Test Results

In the derived HIV dataset, the number of linked EMRs containing an initial positive Western blot result ranged from 1090 to 1427 with all linkage approaches except when two or more linkage keys are needed. The linkage approach which requires two or more linkage keys to match resulted in 257 linked EMRs. The PPV was between 87% and 91% for the first 4 linkage approaches and estimated specificity ranged from 90% to 93%. When fewer EMRs were linked because of the different linkage approaches, both PPV and specificity improved (Table 5).

In the derived hepatitis C dataset, with the first 4 linkage approaches, in excess of 3700 linked EMRs contained an initial positive hepatitis C antibody result, with a drop to 2809 records when two or more linkage keys are needed. The PPV was greater than 98.9% and an estimated specificity was over 99% for all six linkage approaches (Table 5).

## Discussion

### Principal Findings

This paper describes a comprehensive evaluation of a system of probabilistic record linkage using a privacy-preserving software tool within a large-scale health surveillance system. The results showed that this software provides a highly reliable and accurate system for linking routinely collected EMRs through the generation of linkage keys reliant on available identifying information. Optimizing the record linkage involves an appropriate balance between the sensitivity (correctly identifying records belonging to the same person) and specificity (ensuring records that belong to different people are not linked) as well as what will best suit the study design objectives and populations under study without impeding the interpretation of study results.

The high performance of the linkage tool when applied to the relatively small PrEPX gold-standard dataset was related to the data completeness for EMRs in the PrEPX trial compared with the completeness of data in the pathology results gold-standard dataset (Tables 2 and 4). Participants in PrEPX were required to have a Medicare number to be enrolled and have three monthly follow-up visits, which allowed multiple opportunities for the staff at clinics to record any missing identifying information [13]. Where the underlying identifiers are robust and duplication is at a minimum, the probability of missed matches is negligible. In addition, with the PrEPX gold-standard dataset, there was 100% specificity for all linkage approaches, indicating that the linkage tool does not falsely link records in a small sample of EMRs where there was unlikely to be individuals with similar identifying details (name, date of birth, and Medicare number).

When the linkage tool was applied to the larger pathology results gold-standard dataset, sensitivity ranged between 89% and 95% where the linkage approach relied on a single linkage key matching. However, with the approach that requires records to link on two or more linkage key types, sensitivity was reduced to 66%. This is attributable to 22% of EMRs only having a single linkage key type available for linkage, which is mostly because of the Medicare number not being available. The inclusion of laboratory records in the pathology results gold-standard dataset may contribute to a lower sensitivity as a result of patient identifier errors such as mislabeling and recording of laboratory samples [14], compared with the completeness of personal identifiers within clinic EMRs. The final linkage approach where pairs of EMRs were only linked when matched on linkage key type 3 (which does not require the Medicare number) and sex, resulted in 89% sensitivity. This approach was included in the analysis to simulate the performance of the linkage tool when the Medicare number is not available. This is important to evaluate in Australia as a significant proportion of participating sites within ACCESS are funded through jurisdictional governments and do not record patient Medicare numbers [15].

### Limitations

The main challenge in evaluating the GRHANITE Linkage Tool was the development of gold-standard datasets given the deidentified nature of EMRs in ACCESS. Researchers rarely have access to gold-standard datasets on which to perform linkage validation outside large administrative health data sources, and our gold-standard dataset of 86,538 records was comparable with other published studies [16]. The gold standards required records with enough supplementary information for deterministic matching where we could be certain that matches belonged to the same individual and nonmatches belonged to different individuals. Therefore, to generate the gold-standard datasets, there were a limited number of records we could use to accurately calculate sensitivity and specificity of the linkage tool. Although the pathology results gold-standard dataset contained over 80,000 records, one limitation of the evaluation was the inability to identify the correctly unmatched EMRs, which meant specificity could not be directly measured. However, given ACCESS is focused on the surveillance of BBV and STI, we were able to evaluate specificity within the pathology results dataset by examining the concordance of linked test results for HIV and hepatitis C. As expected, linkage specificity was inversely related to sensitivity. In addition, using discordant antibody results, we assumed that any discordant result was attributable to incorrect record linkage as opposed to an error in laboratory test results. However, given the very high sensitivity and specificity of the HIV western blot and antibody tests for HIV and hepatitis C, any testing errors would be minimal. The observed difference in PPV and estimated specificity between the HIV and hepatitis C datasets could be attributed to (1) differences in the sensitivity and specificity of the underlying laboratory tests for HIV and HCV and (2) potentially greater rates of anonymous HIV testing, whereby public laboratories do not require full names for HIV testing [15].

Beyond the false-positive record linkages identified by examining the concordance of linked test results for HIV and hepatitis C, there is potential for other false-positives to occur in cases where individuals share common patient identifiers, such as twins. Given the deidentified nature of ACCESS data, without the actual identifying demographic values, these niche cases cannot be identified. The small impact of these false-positives is not expected to impact the main purpose of public health surveillance using ACCESS. For other research projects that require a lower level of false-positive record linkage, particularly if it is known to contain a high proportion of individuals sharing common patient identifiers, then using a linkage approach that only accepts linkage based on a match of multiple linkage keys would minimize false-positives. In addition, ensuring concordance of other extracted data, such as sex, year of birth, HIV, and hepatitis C testing history, can reduce the level of false-positive record linkages to acceptable levels.

### Conclusions

Evaluating record linkage is an important part of assessing the utility of surveillance and research systems for answering key population-level research questions or for accurately describing population-level trends using linked data. Our findings suggest that the GRHANITE Linkage Tool is appropriate for accurately linking individuals’ episodes of care and underpins the ability for ACCESS to perform privacy-preserving linkage of patient medical records.

## Abbreviations

ACCESS: Australian Collaboration for Coordinated Enhanced Sentinel Surveillance
BBV: blood-borne virus
EMR: electronic medical record
NSW: New South Wales
PPV: positive predictive value
STI: sexually transmissible infection
TAIPAN: Treatment with Antiretrovirals and their Impact on Positive And Negative men
UNSW: University of New South Wales
